# Limited differentiation among *Plasmodium vivax* populations from the northwest and to the south Pacific Coast of Colombia: A malaria corridor?

**DOI:** 10.1371/journal.pntd.0007310

**Published:** 2019-03-28

**Authors:** M. Andreína Pacheco, Kristan A. Schneider, Nora Céspedes, Sócrates Herrera, Myriam Arévalo-Herrera, Ananias A. Escalante

**Affiliations:** 1 Department of Biology/Institute for Genomics and Evolutionary Medicine (iGEM), Temple University, Philadelphia, Pennsylvania, United States of America; 2 Department CB, University of Applied Sciences Mittweida, Mittweida, Germany; 3 Caucaseco Scientific Research Center and Malaria Vaccine and Drug Development Center, Cali, Colombia; 4 Faculty of Health, Universidad del Valle, Cali, Colombia; Johns Hopkins Bloomberg School of Public Health, UNITED STATES

## Abstract

**Background:**

Malaria remains endemic in several countries of South America with low to moderate transmission intensity. Regional human migration through underserved endemic areas may be responsible for significant parasite dispersion making the disease resilient to interventions. Thus, the genetic characterization of malarial parasites is an important tool to assess how endemic areas may connect via the movement of infected individuals. Here, four sites in geographically separated areas reporting 80% of the malaria morbidity in Colombia were studied. The sites are located on an imaginary transect line of 1,500 km from the northwest to the south Pacific Coast of Colombia with a minimal distance of 500 km between populations that display noticeable ethnic, economic, epidemiological, and ecological differences.

**Methodology/Principal findings:**

A total of 624 *Plasmodium vivax* samples from the four populations were genotyped by using eight microsatellite loci. Although a strong geographic structure was expected between these populations, only moderate evidence of genetic differentiation was observed using a suite of population genetic analyses. High genetic diversity, shared alleles, and low linkage disequilibrium were also found in these *P*. *vivax* populations providing no evidence for a bottleneck or clonal expansions as expected from recent reductions in the transmission that could have been the result of scaling up interventions or environmental changes. These patterns are consistent with a disease that is not only endemic in each site but also imply that there is gene flow among these populations across 1,500 km.

**Conclusion /Significance:**

The observed patterns in *P*. *vivax* are consistent with a “corridor” where connected endemic areas can sustain a high level of genetic diversity locally and can restore parasite-subdivided populations via migration of infected individuals even after local interventions achieved a substantial reduction of clinical cases. The consequences of these findings in terms of control and elimination are discussed.

## Author summary

The regional movements of infected individuals that connect suitable transmission areas make malaria resilient to control efforts. Those movements are expected to leave genetic signatures in the parasite populations that can be detected using analytical tools. In this study, the genetic makeups of *Plasmodium vivax* populations were characterized to assess whether the most endemic areas in Colombia were connected. Samples were collected from passive surveillance studies in four locations across an imaginary transect line of 1,500 km from the northwest to the south Pacific Coast of Colombia (South America). Considering the distance, and contrary to expectations, we found weak levels of genetic differentiation between these parasite populations with no evidence indicating that their genetic diversity has been eroded as expected whenever the prevalence of the disease is successfully reduced, e.g., through control programs or environmental changes. Although the sampling lacks the geographic and temporal detail to describe how the dispersion of parasite lineages occurred, the observed patterns are consistent with a series of infected populations that are connected in space by human movements allowing the parasite to diffuse across this 1,500 km transect. This malaria corridor needs to be characterized to achieve elimination.

## Introduction

Malaria elimination is a public health priority. Yet, regardless of a notable reduction in the global malaria burden, more than 40% of the world’s population remains at risk of infection [1]. Although endemic areas of South America historically have had low to moderate malaria transmission [2], they are still a significant challenge to control efforts. Indeed, malaria in South America shows idiosyncratic epidemiologic complexities that involve human behavior (e.g., regional movements), vector ecology, the high prevalence of asymptomatic-subclinical infections, and the presence of *Plasmodium vivax*, a resilient parasite that requires a demanding treatment to eliminate dormant liver parasite forms [3–8]. Moreover, the uncontrolled surge of malaria cases from Venezuela, followed by mass migrations from its underserved endemic areas to neighboring countries, such as Brazil, Colombia, and others, are putting the continent at further risk, threatening to “roll-back” the regional efforts and progress toward elimination [9,10]. Considering all these factors, a characterization of the genetic makeup of malarial parasites in South America will not only provide feedback to the control program, but it also produces a baseline genetic profile of the parasites to further assess the effects of uncontrolled human migration and relocation on malaria incidence.

Parasite genetic studies have focused on detecting deviation from random mating (population structure), a pattern that results from several processes including inbreeding, population expansions, and geographic differentiation due to limited migration [11–15]. Beyond characterizing such patterns, parasite genetic investigations must focus on their correct interpretation in the context of specific questions such as understanding whether movements of infected individuals may connect endemic areas and therefore contribute to monitoring malaria transmission and the persistence of the disease in a given region. Here four *Plasmodium vivax* populations are studied. The sampling sites are located along an imaginary transect line of approximately 1,500 km from the northwest to the south Pacific Coast of Colombia. Those four human populations showed different levels of *P*. *vivax* prevalence [7], and they are separated by a minimal distance of 500 km by road between any two of those populations. Given the noticeable ethnic, economic, and ecological differences between these communities [6–8], parasite migration via human movements between communities is expected to be limited. Thus, the hypothesis that these parasite populations were relatively isolated from each other was tested.

## Materials and methods

### Study sites

We selected four localities with high malaria prevalence but different average annual parasite incidence (API) [7,16]. In all four localities (see Fig 1 in [16]), *P*. *vivax* has shown to be a resilient parasite. These localities are: (i) Tierralta from the Department of Córdoba in the northern area (API ~10.7); (ii) Quibdó (Department of Chocó, API ~25), (iii) Buenaventura (Department of Valle del Cauca, API ~3.1), and (iv) Tumaco (Department of Nariño, API ~6.9) in the southeast area of the Pacific Coast. In Tierralta (~90,000 inhabitants) and Buenaventura (~350,000 inhabitants), the predominant malaria parasite species is *P*. *vivax* (~85% and 75% respectively) while in Quibdó (~100,000 inhabitants) and Tumaco (~160,000 inhabitants) most of the malaria cases are caused by *Plasmodium falciparum* with *P*. *vivax* having a clearly lower prevalence (~30% and 21% respectively). The samples were collected between 2012–2013 in all areas with Buenaventura having an extended sampling (from 2011 to 2015). A complete description of these areas can be found elsewhere [16].

### Ethics statement

A total of 1,328 symptomatic volunteers were passively recruited when visiting the health posts for malaria diagnosis [16,17]. Patients with malaria infection as determined by microscopic examination of Giemsa-stained thick blood smears (TBS) received oral and written explanations about the study and, after free willingness to participate, were requested to sign an informed consent (IC) form previously approved by the Institutional Review Board (IRB) affiliated to the Malaria Vaccine and Drug Development Center (MVDC, Cali-Colombia). The IC from each adult individual or informed assent (IA) from the parents or guardians of children <18 years of age was obtained. Individuals between seven and 17 years of age were asked to sign an additional IA. A trained physician completed a standard clinical evaluation and a physical examination in all symptomatic malaria subjects. All individuals were treated by the local health provider as soon as the blood sample was drawn, using the national antimalarial therapy protocol of the Colombian Ministry of Health and Social Protection (MoH) [16]. Everyone received a unique code number to simplify data collection and identification. Out of the 1,328 patients, 624 samples (47%) from patients infected with *P*. *vivax* were included in this study, and the parasites were genotyped as described below.

### Microsatellite (STRs) genotyping

Total DNA was extracted from the whole blood samples using the PureLink Genomic DNA kit (Invitrogen, USA). Parasite species was confirmed by a Real-time PCR (RT-qPCR) as described elsewhere [18]. Standard *P*. *vivax* DNA positive and negative controls were included in each batch of tests. Genomic parasite DNAs were genotyped by using fluorescently labeled PCR primers that target microsatellite loci (STRs). A set of eight standardized STRs for *P*. *vivax* was used; these loci were selected out of the pool that has been previously explored [11]. In particular, loci MS2, MS5, MS6, MS15 [19] and 14.185, 8.332, 2.21, 3.35 [20]. All PCRs were performed in 15 μL reactions with 2 μL of total genomic DNA, 0.25 mM of each primer, and 7.5 μL of PCR Master Mix (Promega, USA) (it includes 0.05 U/μL of Taq DNA polymerase, 2X reaction buffer, 0.4 mM each dNTP, and 3mM MgCl_2_; Promega, USA). A negative control (nuclease-free dH_2_O) and positive control (a *P*. *vivax* infected blood sample confirmed to be positive by thick blood smear examination) were used. Amplification conditions for PCRs were reported elsewhere depending on the set of primers [19,20]. Fluorescently labeled PCR products were separated on an Applied Biosystems 3730 capillary sequencer and scored using GeneMarker v2.6.7 (SoftGenetics LLC). After the microsatellite pattern was identified across samples in the four populations, all alleles were scored at a given locus if minor peaks were more than one-third the height of the predominant peak. A sample was considered as a single infection if it had only one allele per locus at all the genotyped loci as previously described [11–15,19,20]. The finding of one or more additional alleles at any locus was interpreted as a multiple (polyclonal) infection with two or more haploid genotypes in the same isolate (transmitted by one or several mosquitoes). Missing data (no amplification) were reported by locus but not considered for analyses that require multilocus genotypes, such as haplotype networks.

### The multiplicity of infection and allele frequencies

One of the approaches typically used to compare populations with different transmission intensities is to estimate the average multiplicity of infection (MOI) [21]. MOI is defined as the average number of distinct parasite genotypes concurrently infecting a patient. In this study, MOI was estimated (per loci–ignoring missing data—and on average per population) as the average number of super-infections (neglecting co-infections) using a maximum-likelihood (ML) method that allows estimating profile-likelihood confidence intervals [21]. MOI calculations were stratified by geographical region, where the sample from Buenaventura was split into two groups of samples taken before April 2013 and after April 2013. In addition to MOI, the ML method [21] was employed to estimate allele frequencies at each locus. MOI and allele frequencies were calculated using the R-script provided in [22]. Typically, the MOI estimates from this method have little bias, and the frequency estimates are almost unbiased [22].

To compare the MOI estimates of Tumaco with the other locations a two-sided bootstrap test with the null hypothesis of equality of MOI was performed as follows. For B = 10000 bootstrap samples (consisting of subsamples from Tumaco and the other location of corresponding sample size) the difference in the MOI estimates of Tumaco and the other location were calculated, and *p*-values were obtained by using a bootstrap method [23]. Holm’s multiple correction was applied. Tests were not performed for locus 3.35 as the data for this marker in the Tumaco population was not informative [21,22].

### Population genetic analysis

Under the assumption that patterns in the parasite genetic variation are driven, at least in part, by the regional movement of infected individuals and the local epidemiology [11–13,15], a suite of approaches was used to characterize the genetic variation in the circulating *P*. *vivax* within each population and the differentiation of the parasite populations among the four localities. The genetic diversity within each sampled population was estimated using a series of summary statistics implemented in the Haplotype Analysis software v1.05 [24] where the number of different sampled multilocus genotypes (SMG), the number of unique genotypes (G), the number of private genotypes (PG), and the Nei’s index of genetic diversity (*He*) were estimated [25] on all the multi-locus genotypes that we could unambiguously identify. As usual, *He* was defined as

$$He=[n/(n-1)][1-\sum_{i=1}^{L}p_{i}{}^{2}]$$

where *n* is obtained by taking the sum of identifiable genotypes (phased) over all samples, and *p*_*i*_ is the relative frequency of the *i*-th haplotype (*i* = 1, …, L) in all sampled haplotypes. *He* gives the average probability that a pair of alleles randomly selected from the population is different. For this analysis, complex infections with differences at more than two loci were not included because the haploid genotypes could not be inferred. However, *He* was also calculated per locus. In this case, *p*_*i*_ is the frequency of allele *i*, and *He* is the average probability that a pair of alleles randomly selected from the population is different. For the allele frequencies, the ML estimates [21] were used. Furthermore, non-parametric bias-corrected and accelerated bootstrap confidence intervals were estimated based on 1,000 bootstrap replications using the jackknife estimate for the acceleration factor as described in [23].

To assess the parasite population differentiation between localities, normalized fixation index (*F*st) were also estimated and their significance assessed using a randomization test. A limitation in this kind of analysis is that samples with multiple alleles at more than two loci were not included because the haploid genotypes could not be inferred. Then, an analysis of molecular variance (AMOVA) [26,27] was performed as implemented in GenAIEx version 6.5 [28]. The AMOVA allows comparing the proportion of the parasite genetic variance within and between populations using the PHIPT statistic (analog of the *F*st). Probabilities for the AMOVA statistics were calculated based on individual randomizations.

In order to test that changes in malaria incidence, particularly after deploying interventions, were driven by the introduction or residual presence of a few parasite lineages, pairwise measurements of linkage disequilibrium (LD) were estimated to detect potential clonal expansions. Since many pairs of loci have a different number of alleles, standard measurements of LD will be essentially biased and cannot be adequately compared across loci and populations [29], this is particularly common in microsatellite loci. Thus, conditional asymmetric linkage disequilibrium (ALD) measurements were used since they consider such differences in the number of alleles in each pair of loci [29]. Comparable to other association statistics commonly used to detect LD, ALD estimates go from 0 to 1 with 0 implying total independence and 1 complete linkage. ALD is a measure to compare pairs of loci and requires frequency estimates of two-locus haplotypes of alleles at both loci. To do so, samples with multiple alleles at both loci were disregarded (these were only very few for each two-locus comparison). As a result, it was possible to phase two-locus haplotypes and estimate their frequency using the ML method of [21], as it was done for the allele frequencies of both loci. The estimates were calculated using the R-script provided in [22].

To identify clusters of parasites that could separate the four localities, two methods were used, (i) a Bayesian model-based clustering algorithm that considers admixture as implemented in the Structure v2.3.4 software [30] and (ii) a principal component analyses (PCA) that does not use explicit admixture model (but that is not assumption free). The Bayesian clustering approach assigns genotypes to *K* populations or clusters characterized by a set of allele frequencies at each locus. The observed genetic diversity was evaluated under different *K* values (K = 2 to 15), and each *K* value was run independently 15 times with a burn-in period of 100,000 iterations followed by 100,000 iterations. The admixture model that allows for the presence of individuals with ancestry in two or more of the *K* populations was used in all the analyses [30]. Structure Harvester was used to compute Delta *K* values from Structure [31]. CLUMPP (Cluster Matching and Permutation Program) was used to facilitate the interpretation of population-genetic clustering results [32], and then, distruct v1.1 was used to graphically display the clustering results [33]. The posterior probability for each number of populations or clusters (*K*) was computed, and the *K*-value that better explains the genetic data was an estimate of the number of circulating clusters or populations. Complex infections with differences at more than two loci were not included in this analysis.

Then, PCA was estimated using R on all the samples, but also eliminating alleles that appear less or equal than 5 and 10 times in the whole sample (across all regions) to explore if there was an effect driven by alleles in low frequency. For the PCA, alleles at each locus were coded by 0–1 variables, indicating the absence and presence of alleles, where the number 0–1 variables for each locus coincides with the number of alleles at that locus. This allowed including samples with missing data and samples with multiple infections. Concerning of alleles at low frequencies, alleles that occurred less or equal than 5 or 10 times, were not considered in some of the PCA analyses from the data set by deleting the respective 0–1 variables. This allowed exploring the effect of potential amplification errors without eliminating samples completely. For the PCA only data from Buenaventura collected before April 2013 was included as only this time range of collection is comparable with that of the other regions. Given that, alleles in low frequency could affect MOI, heterozygosity, linkage disequilibrium, and PCA analyses; those calculations were repeated eliminating those alleles that were in low frequency (less than 2%). Although no differences were observed, those results were provided.

Haplotype genealogies found in malaria cases for each locality were inferred for eight microsatellites by using the Global Optimal eBURST algorithm [34], as implemented in PHYLOViZ [35]. Using an extension of the goeBURST rules up to n-locus-variants-level (nLV, where n equals to the number of loci in our dataset: eight), a Minimum Spanning Tree-like structure was drawn to cluster the 386 sequence types (STs) into a clonal complex (CC) based on their multilocus genotypes. This analysis only included single infections and complex infections with differences at only one locus given that the haploid genotypes can be inferred.

## Results

The number of isolates genotyped from each population varied between locations: 258 from Tierralta, 65 from Quibdó, 235 from Buenaventura, and 66 from Tumaco. A description of the samples in terms of single and multiclonal infections, samples with incomplete data, and how they were used on different analyses is reported in S1 Table. Regardless of the differences in sampling, the prevalence of multiple infections showed a reduction north to south. In Tierralta, 84 samples (32.6%) have more than one distinct parasite genotypes concurrently infecting a patient. In the case of Quibdó, Buenaventura, and Tumaco, the number of patients that had infections with more than one lineage in at least one locus was 27 (41.5%), 97 (41.3%), and 31 (47.0%) respectively. However, many of those multiple infections were the result of having more than one allele at only one locus so the lineage-specific genotypes could be easily inferred (S1 Table).

The estimated MOI parameter was relatively low and consistent across all populations sampled (S2, S3 and S4 Tables, S1 Fig). Since samples from Buenaventura encompass a longer period, the MOI between 2011 and 2013 was compared to 2013–2015 showing a slight decline; however, 95% confidence intervals of MOI estimates per locus typically overlapped (see S2 Table). Likewise, there is a slight tendency that MOI was lower in Tumaco when compared to the other localities, but it was also not statistically significant except compared with Buenaventura at markers MS2 and MS15 and with Quibdó at marker 15.

A qualitative inspection of the allele-frequency spectra, as estimated from the maximum likelihood approach (S2 Fig), can provide information on the evolutionary forces acting on the observed variation in the parasite populations; e.g., the patterns suggest that strong bottlenecks did not occur. Here we found that the alleles were shared across all parasite populations; an observation that is consistent with a high degree of relatedness. When examining each parasite population in detail, there was a change in the spectra in Buenaventura between the two periods were compared (2011–2013 versus 2013–2015). Furthermore, Tumaco had the most distinct distribution. This qualitative examination of the allele frequency-spectra provided the first view of patterns that were later identified with more statistically suitable methods (see below).

**Fig 1:**
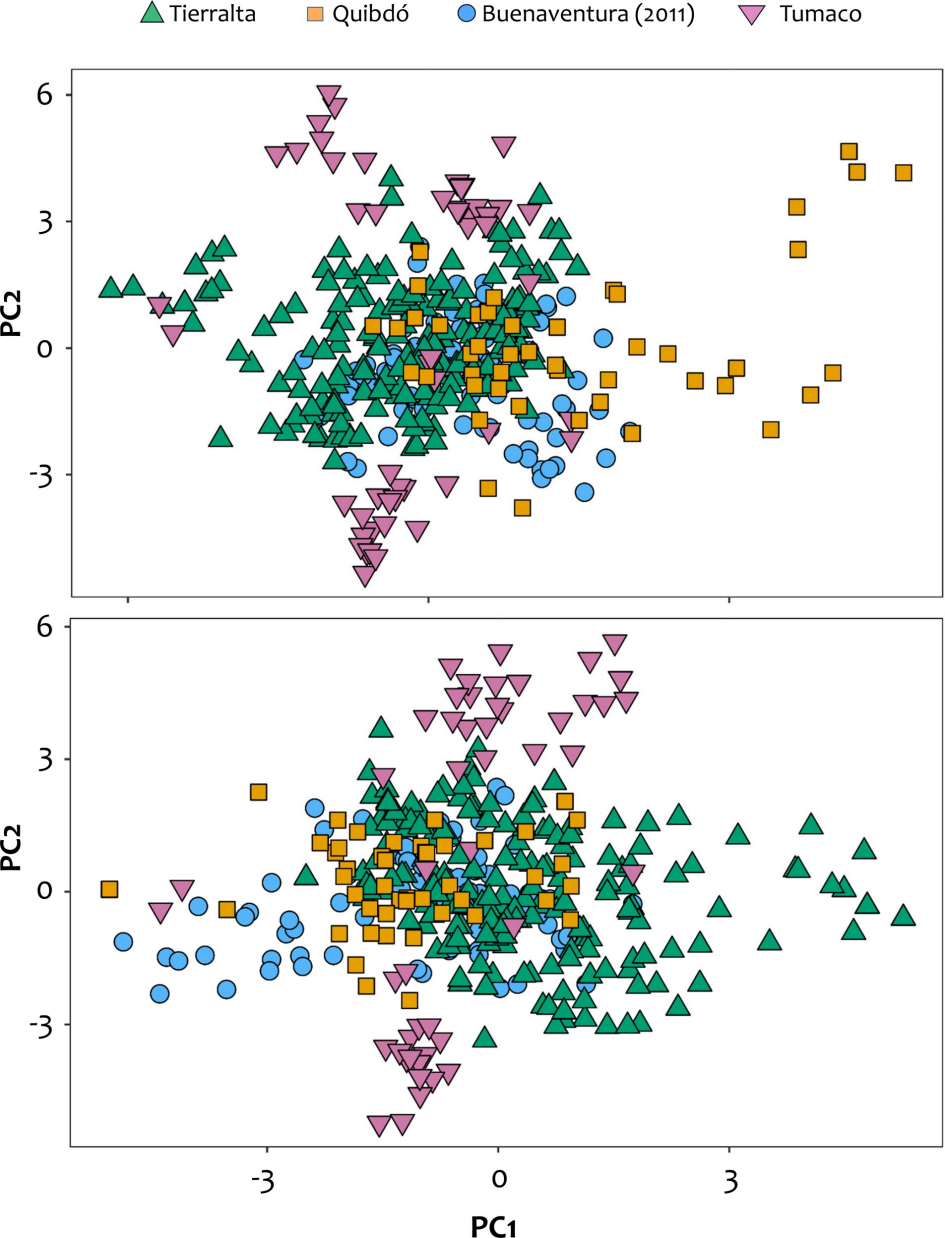
**PCA with alleles with absolute frequency ≤ 5 (top) and ≤ 10 removed (bottom).** Results are almost identical; only the sign of the axes is reversed.

The *P*. *vivax* mean genetic diversity and the relative heterozygosity (per locus and genotype-based) are high in all the sampled populations (Mean *He*: 0.978; S3 Fig, Table 1). This is evident in other summary statistics as well, such as the number of sampled multilocus genotypes (SMG) from the human specimens, the number of distinct genotypes (G), the number of private genotypes (PG), and the Nei’s index of genetic diversity (*He*) (Table 1). This observation seems relatively common in *P*. *vivax* even when the actual loci used to sample the parasite genetic variation differ among studies [15,36–40]. The number of private multi-locus genotypes for *P*. *vivax* in terms of their geographic origin was also very high (Table 1) with only a few multi-locus genotypes being shared between the populations (one genotype shared between Tierralta and Buenaventura). All these observations are consistent with no evidence of a population bottleneck. Likewise, LD is low between loci (S4 Fig) within populations. Because the loci used are physically unlinked, low LD is contrary to the expectations under a bottleneck or clonal expansion scenarios. Tumaco showed the highest LD; this finding was also confirmed by the PCA (Fig 1) where the second principal component divides the Tumaco population into two parts. The other populations have no indication of such a subdivision. Finally, it is worth noting an increase in LD in Buenaventura from 2011–2013 to 2013–2015 (S4 Fig).

**Table 1:** Diversity of multilocus genotypes per Colombian population.

Population	SMG	G	PG	He
Tierralta	218	128	127	0.98
Buenaventura	238	175	174	0.99
Tumaco	71	46	46	0.97
Quibdó	58	38	38	0.97
**Mean**	**146.25**	**96.75**	**96.25**	**0.98**

The number of sampled multilocus genotypes (SMG) from the human specimens, the number of distinct genotypes (G), the number of private genotypes (PG), and the Nei’s index of genetic diversity (He) are shown for each Colombian population. Mean values are shown in bold. All calculations were performed using Haplotype Analysis software v1.05

The normalized *F*st values were significant and relatively high, between 0.10 and 0.23 with Buenaventura and Tierralta being the two populations less differentiated and Tumaco showing the highest *F*st values when compared to the others (Table 2). This result indicates that Tumaco is the most isolated *P*. *vivax* population, which is consistent with geography. Considering that most alleles were shared between populations and that many of those were at low frequency, the interpretation of these *F*st values as strong evidence of population structure requires additional scrutiny. The genetic differentiation between populations was then further explored using an AMOVA (Table 3). Only 5% of the variance was explained by the differences between populations, with 95% within populations indicating that those high *F*st values could be the result of sampling alleles in low frequency.

**Table 2:** Population pairwise *F*st.

	Tierralta	Buenaventura	Tumaco	Quibdó
**Tierralta**	-	-	-	-
**Buenaventura**	0.055	-	-	-
**Tumaco**	0.118	0.130	-	-
**Quibdó**	0.077	0.070	0.117	-

Pairwise fixation index (*F*st) calculated on eight microsatellite loci. *F*st measures of population differentiation due to genetic structure (deviation from random mating). All significant p<0.0000 using a permutation test.

**Table 3:** Analysis of molecular variance (AMOVA).

Source of variation	df	Sum of Squares	Estimated Variance	Percentage of Variance (%)
**Among Pops**	3	21051.162	46.471	5
**Within Pops**	582	539506.872	926.988	95
**Total**	585	560558.034	973.459	100
**Stat**	**Value**	**P (rand > = data)**		
**PhiPT**	0.048	0.001		

PhiPT estimates the proportion of the variance relative to the total. Df; degrees of freedom. The Estimated variance (total and %) are reported.

In the case of the PCA, the first two PCs explained around or less than 10% of the variance (6.5% for all data, 8.5% of alleles with count < = 5, 10.7% of alleles with count < = 10 excluded, Fig 1) if alleles with an absolute frequency less or equal to 10 across all populations were neglected. These differences are expected, e.g., including more alleles with low frequency increases the dimensions of the dataset resulting in less explained variance by the first two principal components. This finding suggests a high relatedness among the populations that were not separated. The first PC was kind of a north-south cline; it separated Tierralta from Buenaventura with Quibdó in the middle, corresponding roughly to the geography. The second PC separated Tumaco from the other population, dividing it into two parts.

**Fig 2:**
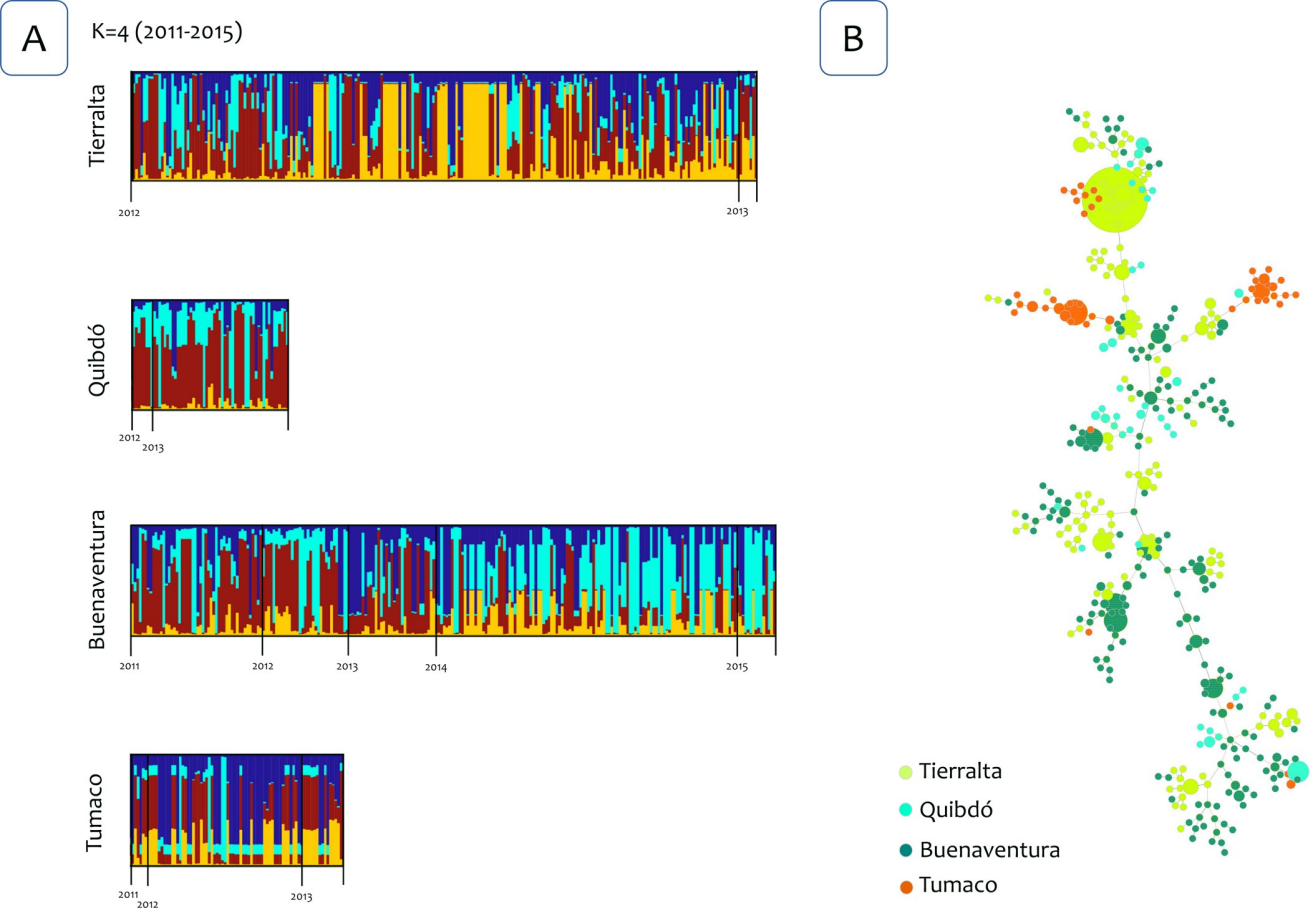
Population structure 2011–2015. (A) Population structure of *P*. *vivax* (*K* = 4) inferred from microsatellite using the STRUCTURE software for four populations from the northwest to the south Pacific Coast of Colombia (years 2011–2015). (B) The tree depicts the relationships among *P*. *vivax* sequence types (ST) at the nLV level (where *n* equals the number of loci in our dataset: eight). Each ST is represented by a circle, and the size of the circle is logarithmically proportional to the number of samples with that particular ST. The color of each circle represents the locality.

**Fig 3:**
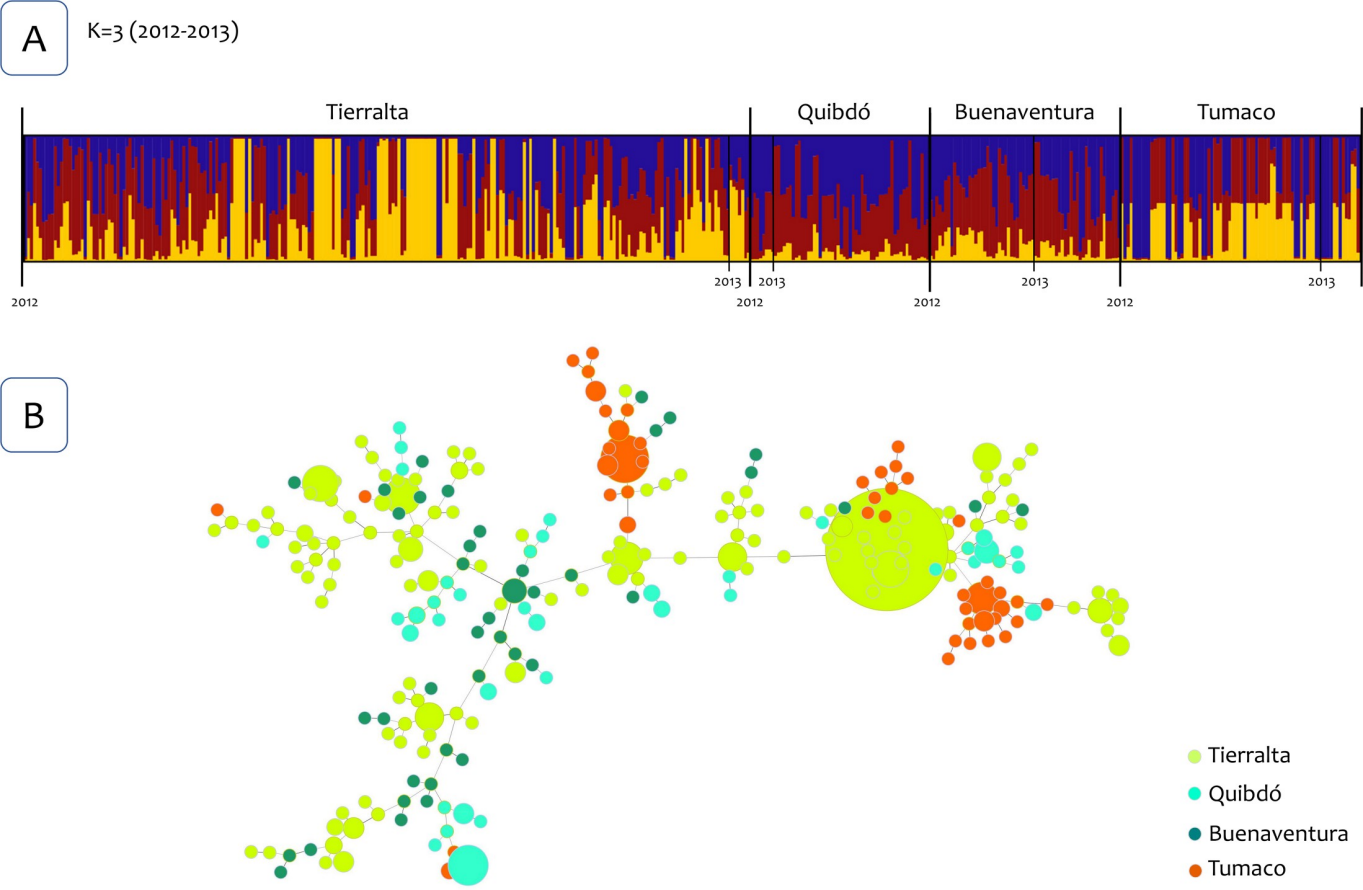
Population structure 2012–2013. (A) Population structure of *P*. *vivax* (*K* = 3) inferred from microsatellite using the STRUCTURE software for four populations from the northwest to the south Pacific Coast of Colombia (years 2012–2013). (B) The tree depicts the relationships among *P*. *vivax* sequence types (ST) at the nLV level (where *n* equals the number of loci in our dataset: eight). Each ST is represented by a circle, and the size of the circle is logarithmically proportional to the number of samples with that particular ST. The color of each circle represents the locality.

**Fig 4:**
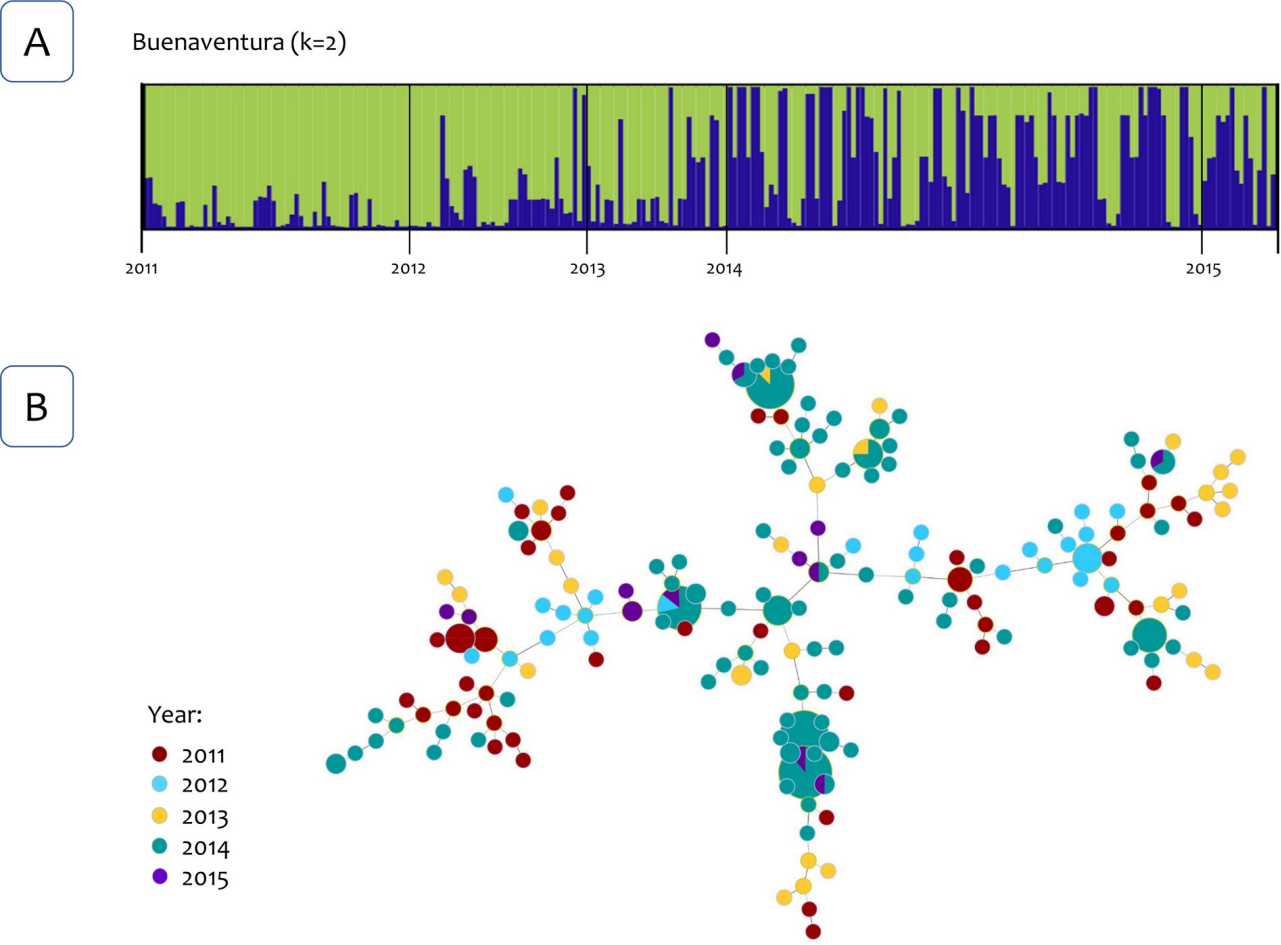
Population structure of Buenaventura parasites. (A) Population structure of *P*. *vivax* for Buenaventura (*K* = 2) inferred from microsatellite using the STRUCTURE software (years 2011–2015). (B) The tree depicts the relationships among *P*. *vivax* sequence types (ST) from Buenaventura at the nLV level (where n equals the number of loci in our dataset: eight). Each ST is represented by a circle, and the size of the circle is logarithmically proportional to the number of samples with that particular ST. The color of each circle represents the years.

The Bayesian clustering using the Structure v2.3.4 software [30] identified four clusters for the four *P*. *vivax* populations (Fig 2A). However, there were not clusters linked to a specific locality or population for all the years that were included in this study (2011–2015). Then, the analysis was repeated using only the samples from 2012 and 2013, and three clusters were identified. However, likewise, no specific clusters were linked to a population indicating admixture (Fig 3A). Interestingly, in both Bayesian cluster analyses, a different pattern in the clusters was identified in Buenaventura after 2013 (Figs 2A and 3A). To further explore this pattern, Structure was run using only samples from Buenaventura from May 2011 to March 2015, and only two clusters were identified (Fig 4A). This analysis also confirmed a change in the clusters in 2014.

Finally, to further examining the relationships between genotypes, haplotypes networks were estimated by the Global Optimal eBURST algorithm [34] using haploid genotypes that could be reconstructed for single infections or those multiclonal infections with highly related multilocus genotypes that differed at one locus only. Three analyses were performed, one included 586 *P*. *vivax* genotypes out of the 624 samples from infected patients from the four locations and all the years included (Fig 2B). Second and third analyses included 447 genotypes from 2012 and 2013 for all localities (Fig 3B), and 248 genotypes from Buenaventura (Fig 4B) respectively. Consistent with the cluster methods, the genotypes did not show clear geographic boundaries.

## Discussion

Malaria parasite populations are expected to exploit habitats that are fragmented. Usually, a mosaic of communities with different levels of malaria prevalence is observed in areas that ecologically and epidemiologically allow transmission. Whereas the mobility of infected individuals is possible, traveling by underserved populations is generally motivated by economic [41] and social factors [42]. In this investigation, the sampled communities are not only separated geographically but are distinct in terms of economic activities and ethnicity [16]. Although towns that are in endemic regions (e.g., Quibdó, Buenaventura, and Tumaco) have urban foci that maintain transmission [43–45], major urban areas (e.g., Bogotá, Cali, and Medellín) that act as economic attractors to the inhabitants from all these sites do not sustain malaria transmission [43]. Thus, a limited level of direct human migration between the sites was expected (e.g., infected individuals moving between any pair of the sampled sites). Considering all these elements, the genetic effects of the parasite population fragmentation between sampled sites were expected to be observed at this geographic scale as result of genetic drift further accelerated by a reduction in the parasite population driven by local deployments of control measurements [11–12,15,38,43,46]. Furthermore, if parasites were re-introduced in a given area or parasite populations were recovering after being diminished by an intervention, clonal expansions resulting from such events would yield significant linkage disequilibrium [11–12].

Contrary to expectations, this study showed that all sampled *P*. *vivax* populations harbored high genetic diversity, a moderate level of genetic differentiation between populations but with several shared alleles, and relatively low linkage disequilibrium within most of the sampled populations. Since the loci sampled are physically unlinked, and contrary to the scenario of a parasite population expansion after a significant reduction in its size, the limited LD observed indicates a low level of inbreeding. This contrasts with previous observations made on *P*. *falciparum* in South America and other *P*. *vivax* populations in the region with high LD (e.g., [11,40,47,48]). Measuring LD using ALD [29] was appropriate since loci exhibited differences in the number of alleles and samples sizes. Furthermore, considering the small number of samples with multiple infections at two loci in every two-locus comparison, the use of ALD allowed incorporating most of the complex infections into the analysis. In addition, using only samples with evident multi-loci genotypes information (e.g., eliminating any sample with multiple infections in two or more loci) biases the estimates.

*F*st values were relatively high (0.10–0.25). However, part of the observed differentiation could be a sampling effect of alleles in low frequency. Indeed, the AMOVA indicated that most of the variation was explained within populations. This is a similar pattern detected in a substantially smaller geographic area in Peru [15]. Nevertheless, in the Peruvian Amazonia, this observation was somehow predictable since the sampled populations shared an economic center (Iquitos) and were interconnected by road separated by a few km (<20 km) [15]. Thus, finding a similar pattern among populations that are separated by at least 500 Km is evidence of an important level of parasite gene flow via human migration. A different approach that allows appreciating these patterns of low differentiation is provided by the PCA and the Structure analyses where individual samples were dispersed, and there were not cluster matching geographic patterns. Furthermore, no evidence of clonal expansions was found (e.g., low LD) indicating a limited effect of the deployed interventions on the parasite genetic diversity.

Although some genetic structures were observed whenever parasite populations were closely examined (e.g., a temporal structure in Buenaventura and two groups within Tumaco), the data available did not support strong geographic isolation between these parasite populations. The temporal changes in the genetic structure in Buenaventura may indicate the introduction of new parasites at the time. However, the low level of LD also indicates that this replacement of parasites was not driven by a few lineages that expanded locally but by the influx of a diverse group of parasites. In the case of Tumaco, it showed the highest *F*st values, and it differs from the other three parasite populations in terms of average LD and genetic diversity. All these observations are consistent with the fact that Tumaco is the most geographically isolated population with the lowest *P*. *vivax* prevalence. However, regardless of these differences, Tumaco shared parasites with the others as indicated by the clustering methods and the haplotype networks (Figs 2–4).

The results presented here are consistent with the existence of a *P*. *vivax* malaria corridor that likely facilitates the persistence of these parasite populations across the sampled imaginary transect line of 1,500 km. In this context, a malaria corridor is defined as spatially connected endemic areas that sustain a high level of genetic diversity regionally and can restore parasite-subdivided populations (“rescue effect”) even after interventions succeeded in reducing transmission locally [49]. Indeed, parasite replacements such as the one documented in Buenaventura after 2013 may be facilitated by human movements and could be common in settings such as the Pacific Coast of Colombia. The identification of the contact zones that effectively sustain the malaria corridor is critical for the long-term success of interventions in Colombia. This is likely the case in other endemic areas of the world where communities are interconnected.

The lack of a suitable temporal and spatial sampling across this transect did not allow describing the dynamic of the parasite dispersion and the identification of the contact zones in the context of the proposed malaria corridor model. However, on the positive side, almost no linked multilocus *P*. *vivax* genotypes were shared between the sampled populations. This suggests a multi-generation gene flow where genotypes are broken by recombination (as detected by microsatellite loci) rather than recently introduced parasite lineages expanding locally. This pattern indicates that regionally coordinated control efforts could increase the fragmentation of the *P*. *vivax* populations making them more susceptible to local extinction (elimination). Furthermore, if it is identified, deploying interventions in such contact zones could be a cost-effective strategy for achieving the elimination goal. A possibility to explore is assessing the effect of mining communities since those may act as catching areas for individuals moving from distant places [41]. It could be speculated that, if these mining areas act as contact zones, deploying interventions there will affect malaria regionally by making parasite populations more fragmented and vulnerable to local elimination if interventions were implemented at a proper temporal and spatial scales. This hypothesis can be tested by genotyping parasites prior to and after interventions in the mining areas. It is worth noticing that patterns suggesting connectivity in South America across long geographic distances have been observed in *P*. *falciparum*, particularly in the Pacific Coast of Colombia and Peru [48,50]. Unlike *P*. *vivax*, *P*. *falciparum* shows a strong LD across populations indicating that such connectivity allows for stable inbreed clones to expand [48]. Nevertheless, the spatial and population structure differences between these two parasite populations in the context of low transmission areas is a matter that should be investigated.

The samples included in this study were mostly from symptomatic patients. However, the importance of asymptomatic carriers in these dynamics can be anticipated [45,51]. Even though genotyping submicroscopic specimens from South America for an extended set of microsatellites is technically difficult with the limited amount of blood collected during malaria surveillance, the handful studies already published with as little as three microsatellites seem to indicate that these patients maintain high genetic diversity [45,52]. Furthermore, there is evidence that they can infect the local vectors [53]. Indeed, asymptomatic individuals could account, at least in part, for the similar level of MOI for *P*. *vivax* infections across settings that indicate a comparable low to moderate transmission in all sites, regardless of differences in malaria morbidity [21]. It is worth noticing that the lack of association of MOI with transmission intensity is consistent with other reports [54]. In addition to asymptomatic patients affecting MOI, other factors should be considered such as the spatial connectivity of an endemic area with unsampled parasite populations and the demographic history of the parasites that determines the detectable lineages given a set of loci (11–16).

Here we provide a static but valuable picture of a complex process critical for malaria control and elimination in a *P*. *vivax* endemic region. Although important in terms of providing a partial description of how parasites disperse in space, studies as the one reported here (as many others) lack the spatial and epidemiological detail required for accurately describing such spatiotemporal dynamics. Nevertheless, this study highlights the need for carefully planned epidemiological investigations that, together with population genetic tools, can accurately model these complex dynamics at an actionable time scale, such as; make inferences from one transmission season to the next.

## Supporting information

S1 Fig MOI and its 95% profile-likelihood confidence intervals. Top: alleles with absolute frequency ≤ 5 among all populations were removed. Bottom: alleles with absolute frequency ≤ 10 among all populations were removed.
(PDF)

S2 Fig Alelle frequencies per microsatellite loci in all Colombian *P*. *vivax* populations. (PDF)

S3 Fig Heterozygosity and its 95% bias-corrected and accelerated non-parametric bootstrap confidence intervals. Top: alleles with absolute frequency ≤ 5 among all populations were removed. Bottom: alleles with absolute frequency ≤ 10 among all populations were removed.
(PDF)

S4 Fig Measurements of conditional asymmetric linkage disequilibrium (ALD) per pair of microsatellite loci for each *P*. *vivax* population. (PDF)

S1 Table Description of isolates genotyped. (PDF)

S2 Table Mean multiplicity of infection (MOI) estimated without alleles removed for the four Colombian population. (PDF)

S3 Table Mean multiplicity of infection (MOI) estimated without alleles removed for Buenaventura. (PDF)

S4 Table Poisson parameter for MOI estimated for each population and Buenaventura alone. (PDF)
