# Supplementary figures and images for: Limited differentiation among Plasmodium vivax populations from the northwest and to the south Pacific Coast of Colombia: A malaria corridor?
Source: PLoS Negl Trop Dis. 2019 Mar 28;13(3):e0007310. doi: 10.1371/journal.pntd.0007310 (PMC6456216; doi:10.1371/journal.pntd.0007310)

● Tierralta ● Quibdó ● Buenaventura (2011-13) ● Buenaventura (2013-15) ● Tumaco

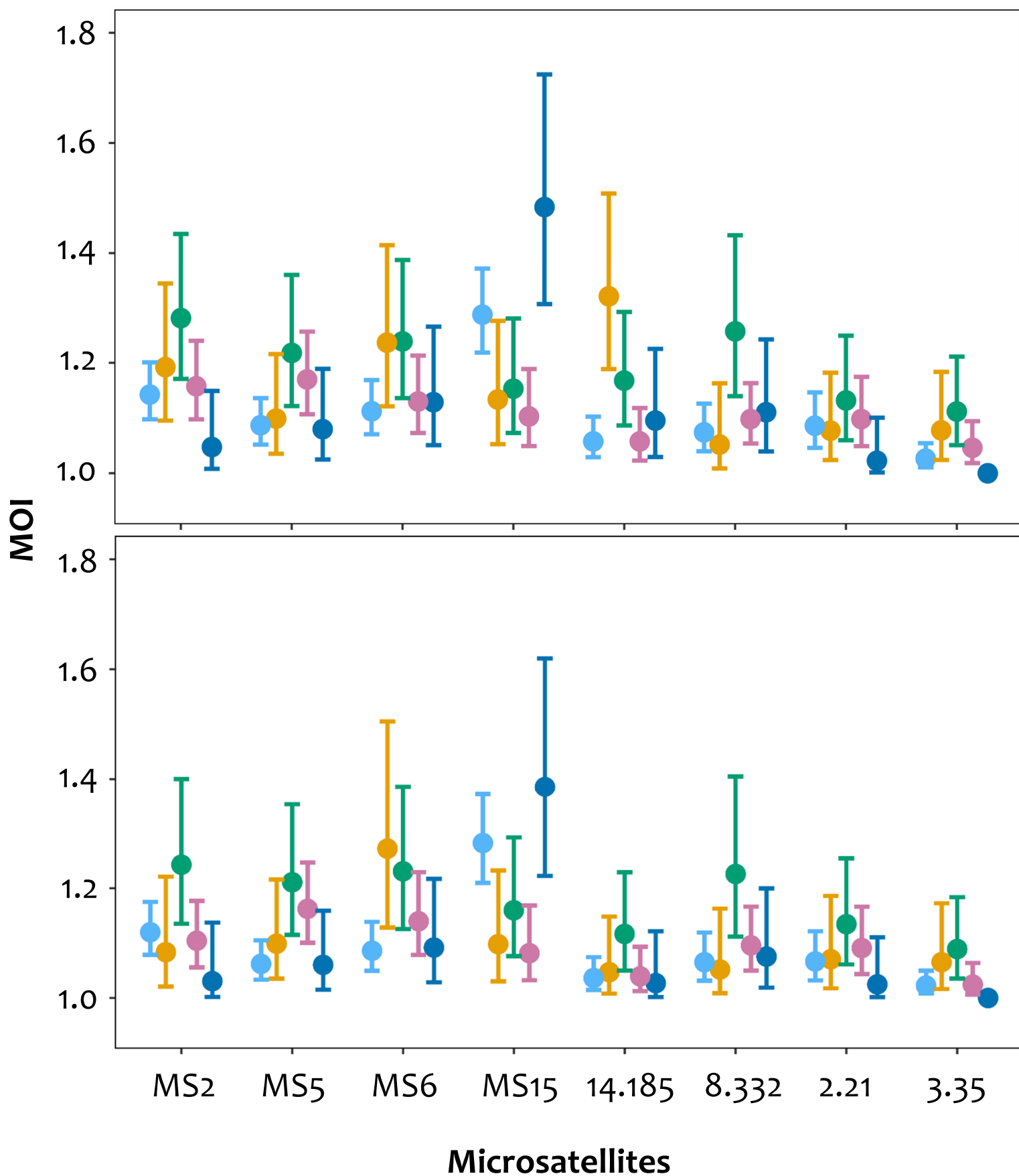

Supplement: S1 Fig — Top: alleles with absolute frequency ≤ 5 among all populations were removed. Bottom: alleles with absolute frequency ≤ 10 among all populations were removed. (PDF) [file pntd.0007310.s001.pdf]

● Tierralta    ● Quibdó    ● Buenaventura (2011-13)    ● Buenaventura (2013-15)    ● Tumaco

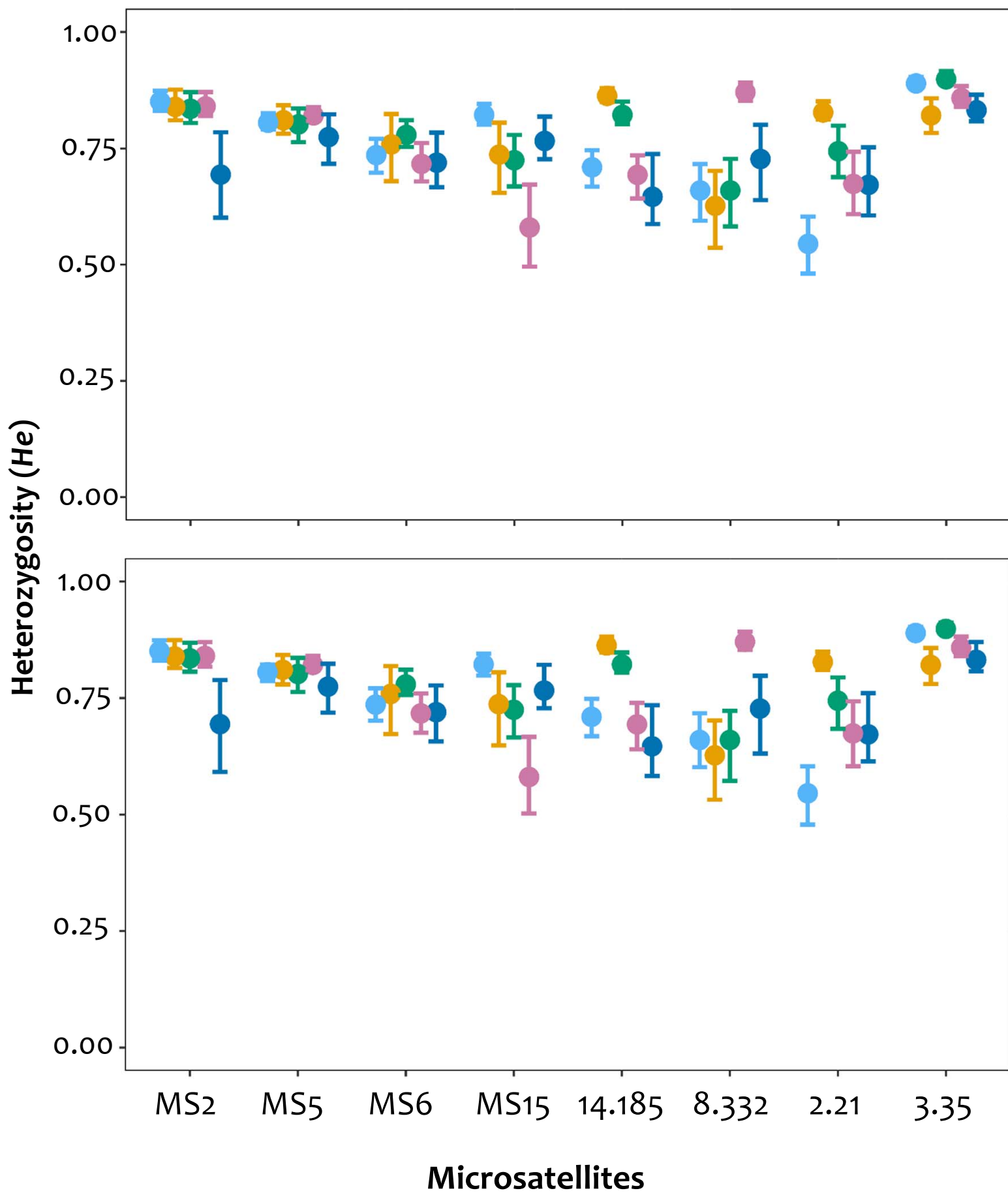

Supplement: S3 Fig — Top: alleles with absolute frequency ≤ 5 among all populations were removed. Bottom: alleles with absolute frequency ≤ 10 among all populations were removed. (PDF) [file pntd.0007310.s003.pdf]
