# Supplementary material for: Limited differentiation among Plasmodium vivax populations from the northwest and to the south Pacific Coast of Colombia: A malaria corridor?
Source: PLoS Negl Trop Dis. 2019 Mar 28;13(3):e0007310. doi: 10.1371/journal.pntd.0007310 (PMC6456216; doi:10.1371/journal.pntd.0007310)
Supplement: S2 Fig — (PDF) [file pntd.0007310.s002.pdf]

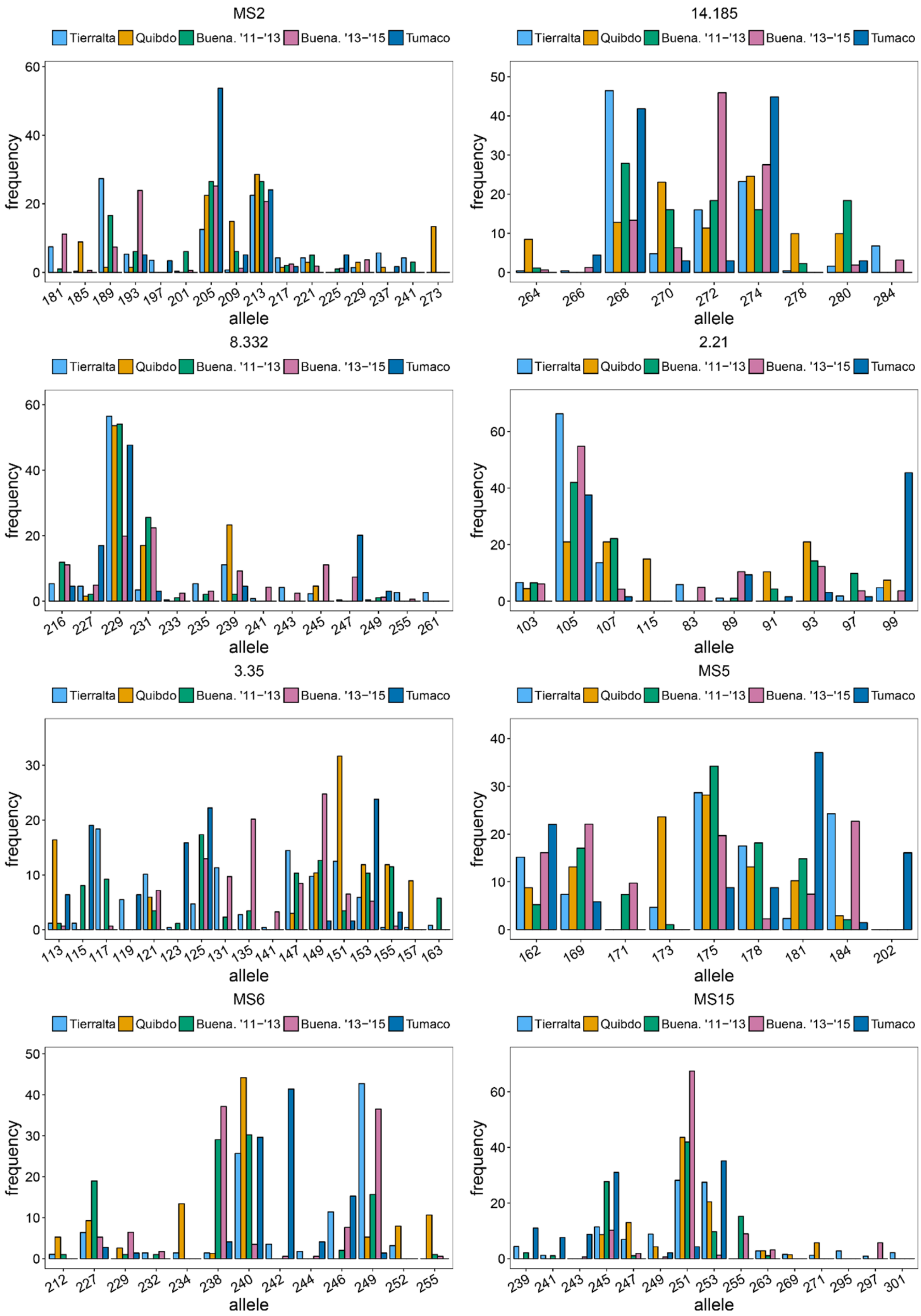

S1A Figure: Allele frequency distribution removing alleles with less than 0.5% frequency.

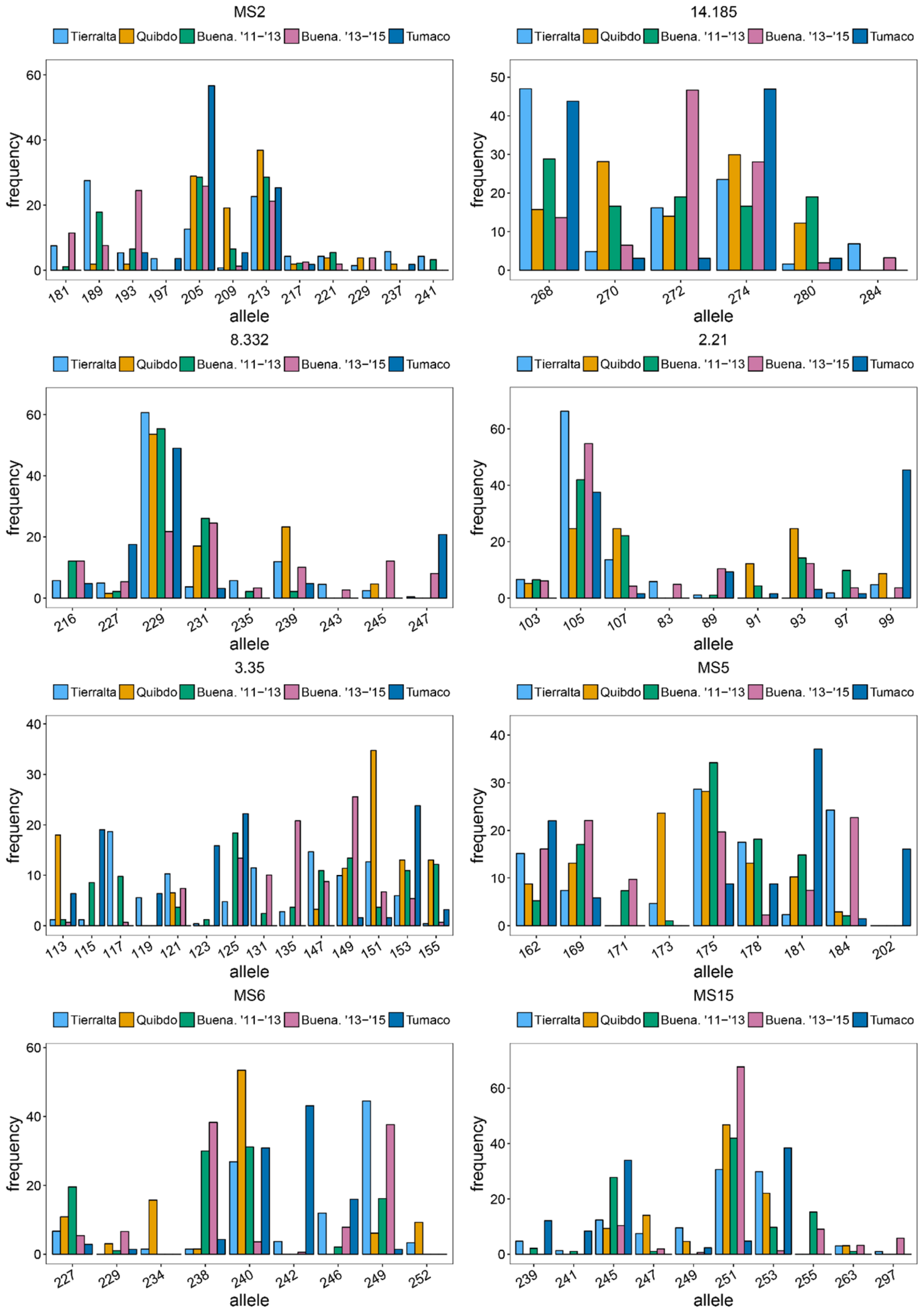

S1B Figure: Allele frequency distribution removing alleles with less than 1% frequency.
