# Supplementary material for: Limited differentiation among Plasmodium vivax populations from the northwest and to the south Pacific Coast of Colombia: A malaria corridor?
Source: PLoS Negl Trop Dis. 2019 Mar 28;13(3):e0007310. doi: 10.1371/journal.pntd.0007310 (PMC6456216; doi:10.1371/journal.pntd.0007310)
Supplement: S1 Table — (PDF) [file pntd.0007310.s005.pdf]

Supporting Information (Supporting Tables)

S1 Table. Description of isolates genotyped.

	**Populations**			
	Tierralta	Quibdó	Buenaventura	Tumaco
Human samples genotyped	258	65	235	66
Samples genotyped without missing data	232	63	226	57
Multiclonal infection (in at least one locus)	84 (32.6%)	27 (41.5%)	97 (41.3%)	31 (47%)
Multiclonal infection (in two loci or more)	50 (19.4%)	15 (23.1%)	39 (16.6%)	9 (13.6%)
Samples used for Structure analyses	208	50	196	57
Samples used for genotype analyses	183	47	182	51
