# Supplementary material for: Limited differentiation among Plasmodium vivax populations from the northwest and to the south Pacific Coast of Colombia: A malaria corridor?
Source: PLoS Negl Trop Dis. 2019 Mar 28;13(3):e0007310. doi: 10.1371/journal.pntd.0007310 (PMC6456216; doi:10.1371/journal.pntd.0007310)
Supplement: S2 Table — (PDF) [file pntd.0007310.s006.pdf]

Supporting Information (Supporting Tables)

S2 Table. Mean multiplicity of infection (MOI) estimated without alleles removed for the four Colombian population.

	Tierralta			Quibdó			Buenaventura			Tumaco		
**Locus**	**MLE**	**95% CI**	**N**	**MLE**	**95% CI**	**N**	**MLE**	**95% CI**	**N**	**MLE**	**95% CI**	**N**
**MS2**	1.143	(1.098, 1.201)	258	1.193	(1.095, 1.344)	63	1.197	(1.141, 1.268)	236	1.048	(1.008 , 1.149)	61
**MS5**	1.087	(1.052, 1.136)	245	1.099	(1.035, 1.216)	63	1.181	(1.127, 1.250)	235	1.080	(1.025, 1.189)	65
**MS6**	1.112	(1.071, 1.169)	258	1.237	(1.121, 1.414)	63	1.163	(1.110, 1.231)	236	1.129	(1.051, 1.266)	66
**MS15**	1.288	(1.219, 1.371)	257	1.134	(1.053, 1.276)	62	1.119	(1.072, 1.184)	236	1.484	(1.306, 1.724)	66
**14.185**	1.058	(1.029, 1.102)	245	1.321	(1.189, 1.508)	63	1.096	(1.057, 1.150)	232	1.096	(1.030, 1.225)	66
**8.332**	1.074	(1.040, 1.126)	247	1.052	(1.009, 1.163)	62	1.137	(1.090, 1.198)	233	1.111	(1.039, 1.242)	63
**2.21**	1.086	(1.046, 1.146)	259	1.077	(1.024, 1.182)	63	1.109	(1.065, 1.168)	236	1.023	(1.001, 1.101)	66
**3.35**	1.027	(1.011, 1.054)	253	1.078	(1.024, 1.184)	63	1.068	(1.038, 1.111)	232	1.000	(NA, NA)	65
**Avg.**	1.109			1.149			1.134			1.121		

Maximum Likelihood Estimation (MLE), 95% of Confidence Interval (CI), and numbers of samples (N) are shown for each locus and each population.
