# Supplementary material for: Limited differentiation among Plasmodium vivax populations from the northwest and to the south Pacific Coast of Colombia: A malaria corridor?
Source: PLoS Negl Trop Dis. 2019 Mar 28;13(3):e0007310. doi: 10.1371/journal.pntd.0007310 (PMC6456216; doi:10.1371/journal.pntd.0007310)
Supplement: S3 Table — (PDF) [file pntd.0007310.s007.pdf]

Supporting Information (Supporting Tables)

S3 Table. Mean multiplicity of infection (MOI) estimated without alleles removed for Buenaventura.

	**Buenaventura**			**Buenaventura 2011-2013**			**Buenaventura 2013-2015**		
**Locus**	**MLE**	**95% CI**	**N**	**MLE**	**95% CI**	**N**	**MLE**	**95% CI**	**N**
**MS2**	1.197	(1.141, 1.268)	236	1.282	(1.171, 1.435)	83	1.158	(1.098, 1.240)	153
**MS5**	1.181	(1.127, 1.250)	235	1.218	(1.122, 1.359)	82	1.170	(1.107, 1.257)	153
**MS6**	1.163	(1.110, 1.231)	236	1.239	(1.136, 1.388)	83	1.130	(1.073, 1.213)	153
**MS15**	1.119	(1.072, 1.184)	236	1.153	(1.073, 1.280)	83	1.103	(1.049, 1.189)	153
**14.185**	1.096	(1.057, 1.150)	232	1.168	(1.086, 1.292)	81	1.058	(1.023, 1.118)	151
**8.332**	1.137	(1.090, 1.198)	233	1.258	(1.140, 1.433)	80	1.098	(1.054, 1.163)	153
**2.21**	1.109	(1.06 , 1.168)	236	1.132	(1.060, 1.249)	83	1.098	(1.049, 1.175)	153
**3.35**	1.068	(1.038, 1.111)	232	1.112	(1.051, 1.211)	80	1.046	(1.018, 1.095)	151
**Avg.**	1.134			1.195			1.108		

Maximum Likelihood Estimation (MLE), 95% of Confidence Interval (CI), and numbers of samples (N) are shown for each locus per years.
