# Supplementary material for: Limited differentiation among Plasmodium vivax populations from the northwest and to the south Pacific Coast of Colombia: A malaria corridor?
Source: PLoS Negl Trop Dis. 2019 Mar 28;13(3):e0007310. doi: 10.1371/journal.pntd.0007310 (PMC6456216; doi:10.1371/journal.pntd.0007310)
Supplement: S4 Table — (PDF) [file pntd.0007310.s008.pdf]

Supporting Information (Supporting Tables)

S4 Table. Poisson Parameter for MOI estimated for each population and Buenaventura alone.

S4A: Poisson Parameter (no alleles removed) for MOI estimated for each population and Buenaventura alone.

	Tierralta			Quibdo			Buenaventura			Tumaco		
locus	MLE	95% CI	N	MLE	95% CI	N	MLE	95% CI	N	MLE	95% CI	N
**MS2**	0.2734	(0.1895,0.3778)	258	0.3633	(0.1849,0.6231)	63	0.3718	(0.2694,0.4957)	236	0.0937	(0.0157,0.2845)	61
**MS5**	0.1693	(0.1017,0.2608)	245	0.1919	(0.0696,0.4044)	63	0.3422	(0.2432,0.4635)	235	0.1565	(0.0491,0.3569)	65
**MS6**	0.217	(0.1379,0.3205)	258	0.4415	(0.2332,0.7387)	63	0.3092	(0.2117,0.431)	236	0.2477	(0.0998,0.4909)	66
**MS15**	0.5288	(0.4092,0.6677)	257	0.2567	(0.1034,0.5089)	62	0.2292	(0.1399,0.3486)	236	0.8491	(0.5607,1.2105)	66
**14.185**	0.1139	(0.0574,0.1982)	245	0.5851	(0.356,0.887)	63	0.1859	(0.1117,0.2861)	232	0.1854	(0.0583,0.4209)	66
**8.332**	0.1454	(0.0782,0.242)	247	0.1022	(0.0171,0.3095)	62	0.2625	(0.1754,0.3731)	233	0.2142	(0.0778,0.4509)	63
**2.21**	0.1681	(0.0905,0.2797)	259	0.1509	(0.0473,0.3444)	63	0.2097	(0.128,0.3192)	236	0.0449	(0.0026,0.1949)	66
**3.35**	0.0529	(0.0211,0.1068)	253	0.1521	(0.0477,0.3471)	63	0.133	(0.0753,0.2138)	232	0	(NA,NA)	65
**AVERAGE**	0.3665			0.2086			0.2239			0.2805		

	Buenaventura			Buenaventura 2011-2013			Buenaventura 2013-2015		
locus	MLE	95% CI	N	MLE	95% CI	N	MLE	95% CI	N
**MS2**	0.3718	(0.2694,0.4957)	236	0.5184	(0.3242,0.7714)	83	0.3008	(0.1893,0.4467)	153
**MS5**	0.3422	(0.2432,0.4635)	235	0.4091	(0.2341,0.6487)	82	0.3233	(0.2062,0.4754)	153
**MS6**	0.3092	(0.2117,0.431)	236	0.4457	(0.2609,0.6952)	83	0.2501	(0.1423,0.3994)	153
**MS15**	0.2292	(0.1399,0.3486)	236	0.2923	(0.1424,0.5162)	83	0.1997	(0.0967,0.3563)	153
**14.185**	0.1859	(0.1117,0.2861)	232	0.3194	(0.1682,0.5366)	81	0.1135	(0.0454,0.2276)	151
**8.332**	0.2625	(0.1754,0.3731)	233	0.4772	(0.2671,0.7677)	80	0.1906	(0.1056,0.3104)	153
**2.21**	0.2097	(0.128,0.3192)	236	0.2533	(0.1172,0.4628)	83	0.1907	(0.0963,0.3309)	153
**3.35**	0.133	(0.0753,0.2138)	232	0.2163	(0.0999,0.3965)	80	0.0914	(0.0365,0.1837)	151
**AVERAGE**	0.2554			0.3665			0.2075		

Maximum Likelihood Estimation (MLE), 95% of Confidence Interval (CI), and numbers of samples (N).

S4B: Mean MOI estimated for each population and Buenaventura alone with alleles with abs. freq. ≤3 removed.

	Tierralta			Quibdo			Buenaventura			Tumaco		
**locus**	**MLE**	**95% CI**	**N**	**MLE**	**95% CI**	**N**	**MLE**	**95% CI**	**N**	**MLE**	**95% CI**	**N**
**MS2**	1.1406	(1.0955 , 1.1986)	255	1.174	(1.0826 , 1.3189)	63	1.1678	(1.1154 , 1.2348)	231	1.0274	(1.0016 , 1.1222)	57
**MS5**	1.0622	(1.0332 , 1.1049)	244	1.099	(1.0352 , 1.2158)	63	1.1729	(1.1196 , 1.2407)	233	1.0605	(1.015 , 1.1589)	65
**MS6**	1.1132	(1.071 , 1.1699)	257	1.2369	(1.1211 , 1.4144)	63	1.1625	(1.1096 , 1.2309)	236	1.129	(1.0507 , 1.2655)	66
**MS15**	1.2675	(1.2007 , 1.3484)	255	1.1338	(1.0526 , 1.2759)	62	1.1177	(1.0685 , 1.1871)	225	1.4577	(1.2836 , 1.6965)	65
**14.185**	1.0527	(1.0252 , 1.0956)	244	1.2343	(1.1238 , 1.4001)	62	1.0798	(1.0448 , 1.1301)	231	1.0955	(1.0294 , 1.2252)	66
**8.332**	1.0689	(1.0356 , 1.119)	246	1.052	(1.0086 , 1.1627)	62	1.1164	(1.0739 , 1.1733)	233	1.1109	(1.0394 , 1.2424)	63
**2.21**	1.0668	(1.0319 , 1.1215)	257	1.0774	(1.0238 , 1.182)	63	1.1051	(1.0623 , 1.1647)	233	1.0248	(1.0014 , 1.1105)	63
**3.35**	1.0225	(1.008 , 1.0485)	251	1.078	(1.024 , 1.1835)	63	1.0593	(1.0316 , 1.1)	229	1	(NA , NA)	63
**AVERAGE**	1.0993			1.1357			1.1227			1.1132		

	Buenaventura			Buenaventura 2011-2013			Buenaventura 2013-2015		
**locus**	**MLE**	**95% CI**	**N**	**MLE**	**95% CI**	**N**	**MLE**	**95% CI**	**N**
**MS2**	1.1678	(1.1154 , 1.2348)	231	1.2474	(1.1432 , 1.3954)	81	1.1307	(1.0761 , 1.2075)	150
**MS5**	1.1729	(1.1196 , 1.2407)	233	1.2112	(1.1148 , 1.3531)	80	1.1625	(1.1005 , 1.2469)	153
**MS6**	1.1625	(1.1096 , 1.2309)	236	1.2394	(1.1361 , 1.3876)	83	1.1303	(1.0729 , 1.213)	153
**MS15**	1.1177	(1.0685 , 1.1871)	225	1.16	(1.0761 , 1.2926)	81	1.095	(1.0404 , 1.1868)	144
**14.185**	1.0798	(1.0448 , 1.1301)	231	1.1258	(1.057 , 1.2372)	80	1.0578	(1.0229 , 1.1181)	151
**8.332**	1.1164	(1.0739 , 1.1733)	233	1.2021	(1.0999 , 1.3611)	80	1.0908	(1.0484 , 1.1536)	153
**2.21**	1.1051	(1.0623 , 1.1647)	233	1.1347	(1.061 , 1.2544)	82	1.0912	(1.0435 , 1.1662)	151
**3.35**	1.0593	(1.0316 , 1.1)	229	1.0982	(1.0419 , 1.1923)	80	1.0398	(1.0142 , 1.086)	148
**AVERAGE**	1.1227			1.1774			1.0998		

Maximum Likelihood Estimation (MLE), 95% of Confidence Interval (CI), and numbers of samples (N).

S4C: Poisson Parameter (alleles with abs. freq. ≤3 removed) estimated for each population and Buenaventura alone.

	Tierralta			Quibdo			Buenaventura			Tumaco		
**locus**	**MLE**	**95% CI**	**N**	**MLE**	**95% CI**	**N**	**MLE**	**95% CI**	**N**	**MLE**	**95% CI**	**N**
**MS2**	0.2692	(0.1854,0.3739)	255	0.3298	(0.1609,0.5817)	63	0.3187	(0.2226,0.4377)	231	0.0543	(0.0031,0.2352)	57
**MS5**	0.1219	(0.0656,0.2029)	244	0.1919	(0.0696,0.4044)	63	0.3279	(0.2304,0.448)	233	0.1187	(0.0298,0.3025)	65
**MS6**	0.2184	(0.1388,0.3225)	257	0.4415	(0.2332,0.7387)	63	0.3092	(0.2117,0.431)	236	0.2477	(0.0998,0.4909)	66
**MS15**	0.4944	(0.3777,0.631)	255	0.2567	(0.1034,0.5089)	62	0.2269	(0.1341,0.3535)	225	0.8078	(0.522,1.17)	65
**14.185**	0.1036	(0.0501,0.1854)	244	0.4369	(0.2381,0.7156)	62	0.1556	(0.0882,0.2497)	231	0.1854	(0.0583,0.4209)	66
**8.332**	0.1348	(0.0704,0.2293)	246	0.1022	(0.0171,0.3095)	62	0.2244	(0.1442,0.3286)	233	0.2142	(0.0778,0.4509)	63
**2.21**	0.1307	(0.0631,0.2339)	257	0.1509	(0.0473,0.3444)	63	0.2033	(0.1221,0.313)	233	0.0493	(0.0028,0.2134)	63
**3.35**	0.0446	(0.0161,0.0955)	251	0.1521	(0.0477,0.3471)	63	0.1163	(0.0626,0.1938)	229	0	(NA,NA)	63
**AVERAGE**	0.1897			0.2578			0.2353			0.2097		

	Buenaventura			Buenaventura 2011-2013			Buenaventura 2013-2015		
**locus**	**MLE**	**95% CI**	**N**	**MLE**	**95% CI**	**N**	**MLE**	**95% CI**	**N**
**MS2**	0.3187	(0.2226,0.4377)	231	0.4596	(0.274,0.7079)	81	0.2508	(0.1485,0.3897)	150
**MS5**	0.3279	(0.2304,0.448)	233	0.3963	(0.2215,0.6388)	80	0.3092	(0.1947,0.4588)	153
**MS6**	0.3092	(0.2117,0.431)	236	0.4457	(0.2609,0.6952)	83	0.2501	(0.1423,0.3994)	153
**MS15**	0.2269	(0.1341,0.3535)	225	0.3045	(0.1485,0.5373)	81	0.1844	(0.0798,0.3528)	144
**14.185**	0.1556	(0.0882,0.2497)	231	0.2419	(0.1119,0.442)	80	0.1135	(0.0454,0.2276)	151
**8.332**	0.2244	(0.1442,0.3286)	233	0.3801	(0.1936,0.6518)	80	0.1765	(0.0952,0.293)	153
**2.21**	0.2033	(0.1221,0.313)	233	0.2583	(0.1196,0.4718)	82	0.1771	(0.0857,0.3159)	151
**3.35**	0.1163	(0.0626,0.1938)	229	0.1904	(0.0826,0.3627)	80	0.0785	(0.0283,0.1674)	148
**AVERAGE**	0.2353			0.3346			0.1925		

Maximum Likelihood Estimation (MLE), 95% of Confidence Interval (CI), and numbers of samples (N).

S4D: Mean MOI estimated for each population and Buenaventura alone with alleles with abs. freq. ≤5 removed.

	Tierralta			Quibdo			Buenaventura			Tumaco		
**locus**	**MLE**	**95% CI**	**N**	**MLE**	**95% CI**	**N**	**MLE**	**95% CI**	**N**	**MLE**	**95% CI**	**N**
**MS2**	1.1296	(1.0862 , 1.1862)	251	1.0784	(1.0242 , 1.1845)	63	1.1562	(1.1054 , 1.222)	227	1.0274	(1.0016 , 1.1222)	57
**MS5**	1.0622	(1.0332 , 1.1049)	244	1.099	(1.0352 , 1.2158)	63	1.1729	(1.1196 , 1.2407)	233	1.0605	(1.015 , 1.1589)	65
**MS6**	1.1087	(1.0674 , 1.1647)	256	1.2165	(1.1069 , 1.3876)	63	1.1615	(1.1081 , 1.2307)	232	1.1082	(1.0385 , 1.236)	66
**MS15**	1.2675	(1.2007 , 1.3484)	255	1.1338	(1.0526 , 1.2759)	62	1.1177	(1.0685 , 1.1871)	225	1.4577	(1.2836 , 1.6965)	65
**14.185**	1.0356	(1.0141 , 1.0725)	243	1.158	(1.0714 , 1.2989)	62	1.0809	(1.0454 , 1.1319)	229	1.0246	(1.0014 , 1.1094)	66
**8.332**	1.0689	(1.0356 , 1.119)	246	1.052	(1.0086 , 1.1627)	62	1.1201	(1.0762 , 1.1788)	228	1.0971	(1.0299 , 1.2295)	60
**2.21**	1.0668	(1.0319 , 1.1215)	257	1.0774	(1.0238 , 1.182)	63	1.1051	(1.0623 , 1.1647)	233	1.0248	(1.0014 , 1.1105)	63
**3.35**	1.0225	(1.008 , 1.0485)	251	1.078	(1.024 , 1.1835)	63	1.0593	(1.0316 , 1.1)	229	1	(NA , NA)	63
**AVERAGE**	1.0952			1.1116			1.1217			1.1000		

	Buenaventura			Buenaventura 2011-2013			Buenaventura 2013-2015		
**locus**	**MLE**	**95% CI**	**N**	**MLE**	**95% CI**	**N**	**MLE**	**95% CI**	**N**
**MS2**	1.1562	(1.1054 , 1.222)	227	1.2326	(1.1322 , 1.3768)	81	1.1191	(1.0667 , 1.1946)	146
**MS5**	1.1729	(1.1196 , 1.2407)	233	1.2112	(1.1148 , 1.3531)	80	1.1625	(1.1005 , 1.2469)	153
**MS6**	1.1615	(1.1081 , 1.2307)	232	1.2374	(1.1322 , 1.3903)	80	1.1318	(1.0737 , 1.2155)	152
**MS15**	1.1177	(1.0685 , 1.1871)	225	1.16	(1.0761 , 1.2926)	81	1.095	(1.0404 , 1.1868)	144
**14.185**	1.0809	(1.0454 , 1.1319)	229	1.1282	(1.058 , 1.2417)	79	1.0586	(1.0232 , 1.1196)	150
**8.332**	1.1201	(1.0762 , 1.1788)	228	1.2134	(1.1054 , 1.3815)	78	1.0932	(1.0496 , 1.1576)	150
**2.21**	1.1051	(1.0623 , 1.1647)	233	1.1347	(1.061 , 1.2544)	82	1.0912	(1.0435 , 1.1662)	151
**3.35**	1.0593	(1.0316 , 1.1)	229	1.0982	(1.0419 , 1.1923)	80	1.0398	(1.0142 , 1.086)	148
**AVERAGE**	1.1217			1.177			1.0989		

Maximum Likelihood Estimation (MLE), 95% of Confidence Interval (CI), and numbers of samples (N).

S4E: Poisson Parameter (alleles with abs. freq. ≤5 removed) estimated for each population and Buenaventura alone.

	Tierralta			Quibdo			Buenaventura			Tumaco		
**locus**	**MLE**	**95% CI**	**N**	**MLE**	**95% CI**	**N**	**MLE**	**95% CI**	**N**	**MLE**	**95% CI**	**N**
**MS2**	0.2489	(0.1677,0.3518)	251	0.1529	(0.0479,0.3488)	63	0.2977	(0.2038,0.4153)	227	0.0543	(0.0031,0.2352)	57
**MS5**	0.1219	(0.0656,0.2029)	244	0.1919	(0.0696,0.4044)	63	0.3279	(0.2304,0.448)	233	0.1187	(0.0298,0.3025)	65
**MS6**	0.21	(0.1318,0.313)	256	0.4056	(0.2066,0.6953)	63	0.3073	(0.2089,0.4307)	232	0.2091	(0.0759,0.4399)	66
**MS15**	0.4944	(0.3777,0.631)	255	0.2567	(0.1034,0.5089)	62	0.2269	(0.1341,0.3535)	225	0.8078	(0.522,1.17)	65
**14.185**	0.0704	(0.0281,0.1417)	243	0.3009	(0.1395,0.5479)	62	0.1577	(0.0894,0.2531)	229	0.0488	(0.0028,0.2114)	66
**8.332**	0.1348	(0.0704,0.2293)	246	0.1022	(0.0171,0.3095)	62	0.2312	(0.1486,0.3385)	228	0.1884	(0.0592,0.4284)	60
**2.21**	0.1307	(0.0631,0.2339)	257	0.1509	(0.0473,0.3444)	63	0.2033	(0.1221,0.313)	233	0.0493	(0.0028,0.2134)	63
**3.35**	0.0446	(0.0161,0.0955)	251	0.1521	(0.0477,0.3471)	63	0.1163	(0.0626,0.1938)	229	0	(NA,NA)	63
**AVERAGE**	0.18196			0.2142			0.2335			0.1845		

	Buenaventura			Buenaventura 2011-2013			Buenaventura 2013-2015		
**locus**	**MLE**	**95% CI**	**N**	**MLE**	**95% CI**	**N**	**MLE**	**95% CI**	**N**
**MS2**	0.2977	(0.2038,0.4153)	227	0.4339	(0.2537,0.6777)	81	0.2295	(0.1305,0.3669)	146
**MS5**	0.3279	(0.2304,0.448)	233	0.3963	(0.2215,0.6388)	80	0.3092	(0.1947,0.4588)	153
**MS6**	0.3073	(0.2089,0.4307)	232	0.4424	(0.2536,0.6997)	80	0.253	(0.144,0.4039)	152
**MS15**	0.2269	(0.1341,0.3535)	225	0.3045	(0.1485,0.5373)	81	0.1844	(0.0798,0.3528)	144
**14.185**	0.1577	(0.0894,0.2531)	229	0.2462	(0.1139,0.4497)	79	0.1149	(0.046,0.2304)	150
**8.332**	0.2312	(0.1486,0.3385)	228	0.4001	(0.204,0.6854)	78	0.1809	(0.0976,0.3002)	150
**2.21**	0.2033	(0.1221,0.313)	233	0.2583	(0.1196,0.4718)	82	0.1771	(0.0857,0.3159)	151
**3.35**	0.1163	(0.0626,0.1938)	229	0.1904	(0.0826,0.3627)	80	0.0785	(0.0283,0.1674)	148
**AVERAGE**	0.2353			0.3340			0.1909		

Maximum Likelihood Estimation (MLE), 95% of Confidence Interval (CI), and numbers of samples (N).

S4F: Mean MOI estimated for each population and Buenaventura alone with alleles with abs. freq. ≤10 removed.

	Tierralta			Quibdo			Buenaventura			Tumaco		
**locus**	**MLE**	**95% CI**	**N**	**MLE**	**95% CI**	**N**	**MLE**	**95% CI**	**N**	**MLE**	**95% CI**	**N**
**MS2**	1.1202	(1.0786 , 1.175)	251	1.0839	(1.0207 , 1.221)	49	1.1478	(1.0974 , 1.2138)	219	1.0307	(1.0017 , 1.1372)	54
**MS5**	1.0622	(1.0332 , 1.1049)	244	1.099	(1.0352 , 1.2158)	63	1.1729	(1.1196 , 1.2407)	233	1.0605	(1.015 , 1.1589)	65
**MS6**	1.0863	(1.0494 , 1.1386)	249	1.2724	(1.1283 , 1.5049)	52	1.164	(1.1089 , 1.2358)	225	1.0921	(1.0284 , 1.2171)	64
**MS15**	1.2827	(1.2096 , 1.3719)	234	1.0984	(1.0303 , 1.2323)	59	1.1107	(1.0632 , 1.1783)	225	1.3858	(1.2224 , 1.6195)	62
**14.185**	1.0365	(1.0144 , 1.0743)	240	1.0474	(1.0078 , 1.1482)	55	1.0653	(1.0338 , 1.1125)	226	1.0273	(1.0016 , 1.1215)	63
**8.332**	1.0655	(1.0313 , 1.1191)	230	1.052	(1.0086 , 1.1627)	62	1.1268	(1.0795 , 1.1906)	213	1.0758	(1.0187 , 1.1996)	59
**2.21**	1.0668	(1.0319 , 1.1215)	257	1.0707	(1.0175 , 1.1859)	54	1.1051	(1.0623 , 1.1647)	233	1.0248	(1.0014 , 1.1105)	63
**3.35**	1.0229	(1.0082 , 1.0495)	247	1.0656	(1.0162 , 1.1725)	58	1.0463	(1.0222 , 1.0839)	222	1	(NA , NA)	63
**AVERAGE**	1.0929			1.0987			1.1174			1.0871		

	Buenaventura			Buenaventura 2011-2013			Buenaventura 2013-2015		
**locus**	**MLE**	**95% CI**	**N**	**MLE**	**95% CI**	**N**	**MLE**	**95% CI**	**N**
**MS2**	1.1478	(1.0974 , 1.2138)	219	1.2431	(1.1352 , 1.4001)	75	1.1046	(1.0556 , 1.1769)	144
**MS5**	1.1729	(1.1196 , 1.2407)	233	1.2112	(1.1148 , 1.3531)	80	1.1625	(1.1005 , 1.2469)	153
**MS6**	1.164	(1.1089 , 1.2358)	225	1.2309	(1.1256 , 1.386)	78	1.1401	(1.0783 , 1.2292)	147
**MS15**	1.1107	(1.0632 , 1.1783)	225	1.16	(1.0761 , 1.2926)	81	1.0819	(1.0323 , 1.1685)	144
**14.185**	1.0653	(1.0338 , 1.1125)	226	1.117	(1.0498 , 1.2291)	77	1.0398	(1.0123 , 1.0931)	149
**8.332**	1.1268	(1.0795 , 1.1906)	213	1.2263	(1.1117 , 1.4048)	76	1.0963	(1.0497 , 1.1664)	137
**2.21**	1.1051	(1.0623 , 1.1647)	233	1.1347	(1.061 , 1.2544)	82	1.0912	(1.0435 , 1.1662)	151
**3.35**	1.0463	(1.0222 , 1.0839)	222	1.0895	(1.0353 , 1.1837)	76	1.0246	(1.0061 , 1.0641)	145
**AVERAGE**	1.1174			1.1766			1.0926		

Maximum Likelihood Estimation (MLE), 95% of Confidence Interval (CI), and numbers of samples (N).

S4G: Poisson Parameter (alleles with abs. freq. ≤10 removed) estimated for each population and Buenaventura alone.

	Tierralta			Quibdo			Buenaventura			Tumaco		
**locus**	**MLE**	**95% CI**	**N**	**MLE**	**95% CI**	**N**	**MLE**	**95% CI**	**N**	**MLE**	**95% CI**	**N**
**MS2**	0.2316	(0.1533,0.3316)	251	0.1633	(0.0411,0.4136)	49	0.2823	(0.1888,0.4009)	219	0.0608	(0.0035,0.263)	54
**MS5**	0.1219	(0.0656,0.2029)	244	0.1919	(0.0696,0.4044)	63	0.3279	(0.2304,0.448)	233	0.1187	(0.0298,0.3025)	65
**MS6**	0.1679	(0.0973,0.2654)	249	0.5029	(0.2465,0.8819)	52	0.3118	(0.2104,0.4394)	225	0.1789	(0.0562,0.4066)	64
**MS15**	0.5204	(0.3935,0.6696)	234	0.1907	(0.0599,0.4334)	59	0.2138	(0.1239,0.3376)	225	0.6923	(0.4161,1.0564)	62
**14.185**	0.0721	(0.0288,0.145)	240	0.0934	(0.0157,0.2831)	55	0.1279	(0.0668,0.2172)	226	0.0542	(0.0031,0.2339)	63
**8.332**	0.1282	(0.0619,0.2294)	230	0.1022	(0.0171,0.3095)	62	0.2438	(0.155,0.3596)	213	0.1479	(0.0372,0.3757)	59
**2.21**	0.1307	(0.0631,0.2339)	257	0.1383	(0.0347,0.3514)	54	0.2033	(0.1221,0.313)	233	0.0493	(0.0028,0.2134)	63
**3.35**	0.0455	(0.0164,0.0974)	247	0.1285	(0.0323,0.3272)	58	0.0912	(0.044,0.1634)	222	0	(NA,NA)	63
**AVERAGE**	0.1773			0.1889			0.2253			0.1628		

	Buenaventura			Buenaventura 2011-2013			Buenaventura 2013-2015		
**locus**	**MLE**	**95% CI**	**N**	**MLE**	**95% CI**	**N**	**MLE**	**95% CI**	**N**
**MS2**	0.2823	(0.1888,0.4009)	219	0.4522	(0.2592,0.7155)	75	0.2023	(0.1093,0.3352)	144
**MS5**	0.3279	(0.2304,0.448)	233	0.3963	(0.2215,0.6388)	80	0.3092	(0.1947,0.4588)	153
**MS6**	0.3118	(0.2104,0.4394)	225	0.431	(0.2414,0.6926)	78	0.2683	(0.1528,0.4279)	147
**MS15**	0.2138	(0.1239,0.3376)	225	0.3045	(0.1485,0.5373)	81	0.1596	(0.0639,0.32)	144
**14.185**	0.1279	(0.0668,0.2172)	226	0.2254	(0.0981,0.4277)	77	0.0786	(0.0245,0.1808)	149
**8.332**	0.2438	(0.155,0.3596)	213	0.4228	(0.2157,0.7232)	76	0.1868	(0.0978,0.3161)	137
**2.21**	0.2033	(0.1221,0.313)	233	0.2583	(0.1196,0.4718)	82	0.1771	(0.0857,0.3159)	151
**3.35**	0.0912	(0.044,0.1634)	222	0.174	(0.0698,0.3473)	76	0.0488	(0.0122,0.1255)	145
**AVERAGE**	0.2253			0.3331			0.1788		

Maximum Likelihood Estimation (MLE), 95% of Confidence Interval (CI), and numbers of samples (N).
